# Differences in Situational Patterns During Change of Direction Movements Greater than 90° in Youth Male and Female Soccer Players

**DOI:** 10.5114/jhk/169524

**Published:** 2023-09-05

**Authors:** Aki-Matti Alanen, Eric S. Gibson, Meghan Critchley, Lauren C. Benson, Matthew J. Jordan, Reed Ferber, Kati Pasanen

**Affiliations:** 1 Integrative Neuromuscular Sport Performance Laboratory, Faculty of Kinesiology, University of Calgary, Calgary, Alberta, Canada.; 2 Sport Injury Prevention Research Center, Faculty of Kinesiology, University of Calgary, Calgary, Canada.; 3 Tonal Strength Institute, San Francisco, USA.; 4 Faculty of Kinesiology, University of Calgary, Calgary, Canada.; 5 McCaig Institute for Bone and Joint Health, University of Calgary, Calgary, Canada.; 6 Running Injury Clinic, Calgary, Canada.; 7 Faculty of Nursing, Cumming School of Medicine, University of Calgary, Calgary, Canada.; 8 Alberta Children’s Hospital Research Institute, University of Calgary, Calgary, Canada.; 9 Tampere Research Center of Sports Medicine, UKK Institute, Tampere, Finland.

**Keywords:** youth soccer, agility, athlete testing, playing position

## Abstract

Change of direction (COD) maneuvers in soccer create tactical advantages, but also expose the player to an increased risk of injury. COD ability is commonly tested with pre-planned drills including cuts greater than 90°. These tests do not take into consideration positional differences players encounter during games. This case-series study used principal component analysis (PCA) to examine situational differences during COD movements between playing positions in youth soccer games. For each of the four teams included (26 females, 27 males), one game was analyzed using video-analysis. Two independent reviewers identified situational patterns and a PCA was used to examine differences between playing positions. Three principal components explained 89% of the variation in the data and were categorized as the total quantity of CODs, attacking/goal-scoring and defensive reacting types of CODs. One-way ANOVA on the individual principal component (PC) scores showed significant differences (p < 0.05) between centre midfielders, goalkeepers, and centrebacks in the quantity of CODs (PC1), and between wingers and fullbacks and centre backs in attacking/goal-scoring CODs (PC2), whereas PC3 was not different between playing positions. Differences between playing positions suggest that training and testing protocols in soccer could be enhanced to better match the individual and playing position-based needs.

## Introduction

During a game, soccer players perform accelerations, decelerations and change of direction (COD) movements with various speeds and in different tactical situations (Bloomfield et al., 2007). Effective COD movement in games requires not only speed, balance, agility and power, but also the ability to perceive and react to movement of the ball and other players (Bloomfield et al., 2007; Reilly et al., 2000). COD ability has been connected with talent identification in youth soccer, with elite players performing faster in COD tests when compared to sub-elite players (Gil et al., 2007; Reilly et al., 2000). The number of high intensity actions in soccer has been steadily increasing in the last years and fast changes of running directions while anticipating and making decision account for about 90% of these actions (Andrzejewski et al., 2013; Barnes et al., 2014). Observation of situation-specific CODs during games is warranted to develop player evaluation methods further, for purposes of performance enhancement, injury prevention and rehabilitation.

Previous studies have looked at player movement characteristics during games, but the definition of “change of direction” movement varies within the literature (Brughelli et al., 2008; Sheppard and Young, 2006; Young et al., 2021), resulting in different outcomes for the quantity of CODs in a game (Bloomfield et al., 2007; Morgan et al., 2022; Nedelec et al., 2014). Different situational patterns (e.g., direction of movement, playing position, speed, ball possession, competition) influence the number and the type of CODs performed, and different training configurations have been shown to affect results in traditional COD tests (De Villarreal et al., 2023). Studies analyzing inter-limb asymmetries have concluded that the COD deficit revealed greater side-to-side asymmetries compared to COD times (Arboix-Alió et al., 2021). However, the majority of testing protocols to evaluate players’ performance or readiness to play after an injury include COD tests that are performed as straight sprint(s) with pre-planned cutting over 90° regardless of the playing position (Alanen et al., 2021; Ardern et al., 2016; Dos’Santos et al., 2020; Loturco et al., 2018; Nimphius et al., 2018; Papla et al., 2022). These tests have been criticized due to the bias towards assessing straight-line sprinting speed, which has been shown to represent an independent performance capacity that is separate from COD performance (Ari et al., 2021; Nimphius et al., 2018; Willberg et al., 2023). Additionally, differences by the playing position might affect player loading during games as previous studies have shown that runs with a COD might increase energy expenditure (Ari et al., 2021). Identification and quantification of COD patterns under currently used COD testing conditions are needed to understand how players behave in games according to their playing position and to determine if there is a need for individualization of COD assessments.

Differences between playing positions and COD situational patterns during games in youth soccer have not been studied thoroughly. Thus, an analysis of positional and situational differences in COD movements is warranted to develop a better understanding of the differences of physical and tactical demands across playing positions. This can provide important information for player testing and training related to sport performance and sport injury. The aim of this case-series study was to examine and describe situational differences in 90° cuts, 135° cuts and 180° pivot turns in games with respect to different playing positions in youth female and male soccer players.

## Methods

### Study Design and Participants

Four elite U16-U17 youth soccer teams (2 male and 2 female teams) from a local soccer club in Calgary, Alberta, were invited to participate in this cross-sectional study. Team recruitment occurred prior to the outdoor soccer season (May–October). Final participation was based on the signed mature minor consent of each player and their parent/guardian. All players with a minimum of 30 minutes (1/3 of the game) of playing time were included. Thirty minutes were determined to be the minimum time to quantify COD movements based on the information from previous studies on playing intensity and performance. As playing time increases, players will cover smaller distance, perform fewer high-intensity actions and situate further away from each other, especially on the second half of play (Barros et al., 2007; Caetano et al., 2019). Reductions in distance covered will start during the first half, while the first 15 minutes of play include most high-intensity running and highest intensities have been recorded during first 15–30 minutes (Bradley et al., 2009; Coelho et al., 2016; Mohr et al., 2003). If a player could not be recognized from the video, due to the camera angle or other obstructions of the view, they were excluded from the analysis. Ethical approval was granted by the Conjoint Medical Ethics Committee (REB19-0428, approval date: 24 April 2019).

### Procedures

Information on the player’s age, training experience, playing position, and previous lower extremity injuries (within 12 months from baseline testing) were collected with a self-reported baseline questionnaire. The following playing positions were identified: goalkeeper (GK), centre back (CB), full back (FB), centre midfield (CM), winger (W) and striker (S). All teams used a similar formation (4-5-1) on the field, which was the base for the playing position identification. Additionally, all teams played against another top-level (Tier 1) opponent in a league-game. Player height (cm) was measured using a portable height measurement unit (Seca GmbH, Seca 217) and body mass (kg) was evaluated with a portable standard electronic scale (Seca GmbH, Seca 437). Both body height and mass measurements were completed barefoot and wearing a t-shirt and shorts. Game data were collected by videorecording one game per team with two 4K cameras (Sony, FDR-AX53, 120fps). The cameras were placed on tripods and covered opposite sides of the field. Players were identified based on their jersey numbers.

### COD Identification

CODs were identified when a player ran forward, changed direction while running (plant and cut) and then continued the run without stopping in the new direction. CODs that included running backwards, side-shuffling or a combination of these with straight running were not considered. Situational patterns were recorded for each identified COD and included the following: ball possession (player/team), running speed before COD movement (low/moderate or fast), contact with other players during COD movement, the side (right or left) and the angle of COD movement (90° cut/135° cut/180° pivot turn). Ball possession (player) was identified if the player was dribbling or had control over the ball after receiving a pass. Ball possession (team) was identified if a player from the same team had control over the ball or the ball was being passed between players. Body contacts were identified from other players to any body part during or right before COD, and a challenge was identified as an action from an opposing player that forced a perception-reaction response. The turning direction was identified as left or right and running speed was classified into two categories: fast or low/moderate. Fast running speed was identified when a player was chasing the ball/opponent or was being chased when with ball possession. Low/moderate speed was identified when a player was jogging and/or not pressing/being pressed. All COD movements were identified using video-analysis software (Dartfish Live S) and a soccer-specific tagging panel designed for identifying situational patterns.

Two independent reviewers (A.-M.A., E.S.G.) completed the video tagging task. Reviewer 1 had over ten years of experience in soccer video-analysis through coaching and was responsible for training reviewer 2. After the training period, both reviewers identified the COD movements for seven players from one team (U16 boys) separately to determine interrater reliability and control for misclassification bias. The number of players for the initial identification was determined on playing time; all those athletes played more than 2/3 of the game. The interrater reliability was excellent, with ICC (3,k) > 0.95 for all situations.

**Figure 1:**
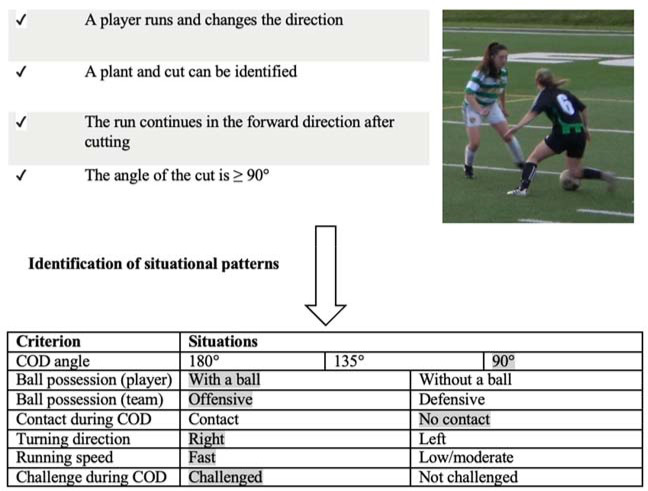
Flowchart of COD and situational pattern identification, with examples (shadings).

After the initial identification and confirmation of agreement, the reviewers independently identified the rest of the games and players (two teams per reviewer). The criteria for identification of each situational pattern are presented in Figure 1. To account for unequal playing time, COD situation counts were expressed per 45 minutes (half of a game) of playing time.

### Statistical Analysis

Means with standard deviations (SD) were used to describe continuous data, and frequency tables were used for categorical variables. Principal Components Analysis (PCA) was conducted to extract and visualize the information from the original set of situational variables. Before applying PCA to the data, the suitability of the dataset was inspected using the Bartlett’s test for sphericity and the Kayser-Meyer-Olkin (KMO) test for sampling adequacy. The *p*-value for the Bartlett’s test was <0.001 and the KMO values for all the variables were over 0.5 (range from 0.69 to 0.87) indicating that the dataset was suitable for PCA.

Before performing PCA, the dataset was first standardized to standard deviation of one and zero mean, because the means and standard deviations for counts between the playing positions were largely different. After Varimax rotation, three principal components explained 89% of the variation between the playing positions. Based on the systematic review by Rojas-Valverde et al. (2020) there are varying considerations for factor retention, with the most common consideration being eigenvalues of >1. However, there are many methods for determining the number of relevant components and most fail to determine the optimal number (Ferré, 1995). In this study, a method used by Benson et al. (2019) was applied, where components that contributed modes of variation greater than an equivalently sized input matrix of normally distributed randomly generated numbers, were decided to be retained.

PCA was conducted with R software (R-foundation), using FactoMineR and factoextra packages. Quality criteria for reporting the results of PCA proposed by Rojas-Valverde et al. (2020) were used as the basis for reporting, to increase understanding and future applicability. One-way analysis of variance (ANOVA) was used to determine whether playing positions showed statistically significant differences based on individual PC scores. Statistical significance was set beforehand as *p* < 0.05 and the Tukey’s test was used for post-hoc analysis.

## Results

Sixty-two players entered the study and 58 played at least 30 minutes in the recorded game. Six players were excluded from the analysis due to inability to identify COD movements (i.e., a player was too far away, other players obstructed the view). Descriptive characteristics for players who completed the study (n = 52) are presented in Table 1.

**Table 1:** Players’ characteristics.

	Males (n = 25)	Females (n = 27)
**U17, n**	11	13
**U16, n**	14	14
**Age**	16.2 (0.7)	16.3 (0.7)
**Body height**	175.7 (9.4)	166.2 (5.8)
**Body mass**	66.9 (5.4)	59.6 (8.7)
**Soccer training exposure (years)**	10.8 (1.7)	10.4 (1.8)
**Playing time / game**	65 (18)	68 (20)
**PLAYING POSITION**		
**GK, n**	2	2
**CB, n**	4	4
**FB, n**	5	4
**CM, n**	5	8
**W, n**	6	6
**S, n**	3	3
**Lower extremity injury within last 12 months**		
**Yes, n**	10	13
**No, n**	14	11
**Injury report missing, n**	1	3

### Principal Component Analysis

Eigenvalues for the three PCs were 10.9 (PC1), 1.5 (PC2) and 1.14 (PC3). The first principal component explained most of the variation (72.4%). All 15 variables correlated with PC1 at a significance level of *p* < 0.0001, indicating that PC1 was related to the quantity of CODs regardless of situational patterns. All factor loadings except for fast speed for PC1 were >0.75, which is usually considered strong loading (Rojas-Valverde et al., 2020). Eight variables correlated with PC2 (*p* < 0.05), suggesting related relationship with attacking situations (fast, 90°, CODs to the right). Seven variables correlated with PC3 (*p* < 0.05), which indicated that PC3 was related to defensive situations requiring a response to opposition movements (fast, without a ball, contact, defensive). However, factor loading for PC2 and PC3 was between 0.3 and 0.7, and was considered weak (0.3–0.49) or moderate (0.5–0.75) loading (Rojas-Valverde et al., 2020).

The results of ANOVA and *p*-values adjusted for multiple comparisons are presented in Tables 2 and 3. PC1 and PC2 showed significant differences between the playing positions (*p* < 0.05), whereas PC3 did not. Post-hoc (Tukey’s test) analysis revealed significant (*p* < 0.05) differences when comparing center midfielders with goalkeepers or center backs in PC1. Differences for PC2 were found between wingers and center midfielders as well as wingers and full backs. Table 3 shows results of the post-hoc test (Tukey’s test) for the comparisons that were statistically significant (*p* < 0.05).

**Table 2:** ANOVA for principal components 1–3.

PC1	Degrees of freedom	Sum of squares	Mean square	F-value	*p*-value
**Position**	5	153.7	30.745	3.445	0.01*
**Residuals**	45	410.5	8.924		
**PC2**					
**Position**	5	20.07	4.015	3.208	0.01*
**Residuals**	45	57.57	1.252		
**PC3**					
**Position**	5	0.43	0.0864	0.068	0.997
**Residuals**	45	58.58	1.2734		

* = statistical significance p < 0.05

**Table 3:** Tukey’s multiple comparison of means for playing positions.

Positions	Difference	Lower	Upper	*p*-adjusted
**PC1**				
**CM-GK**	5.33	0.25	10.41	0.03*
**CM-CB**	4.19	0.21	8.19	0.03*
**PC2**				
**W-FB**	1.45	−0.02	2.92	0.05*
**W-CM**	1.62	0.29	2.95	0.01*

* = statistical significance p < 0.05

Abbreviations: COD = Change of direction, 180 ° = *180* *° pivot turn; 135* *° = 135* *° cut; 90* *° = 90* *° cut; GK* = Goalkeeper; CB = Centre back; FB = Fullback; CM = Centre midfielder; W = Wide midfielder/Winger; S = Striker

Center midfielders performed more CODs regardless of the situational pattern, compared to goalkeepers and central defenders (PC1, *p* < 0.05). Wingers performed fast CODs, with 90° cuts and turning to their right more often than centre midfielders and fullbacks (PC2, *p* < 0.05). Fullbacks and centre midfielders performed more 180° pivot turns, 135° cuts, low/moderate speed and to their left, than wingers (PC2, *p* < 0.05). Centre backs and goalkeepers completed the least amount of any types of CODs during a game (PC1).

**Figure 2:**
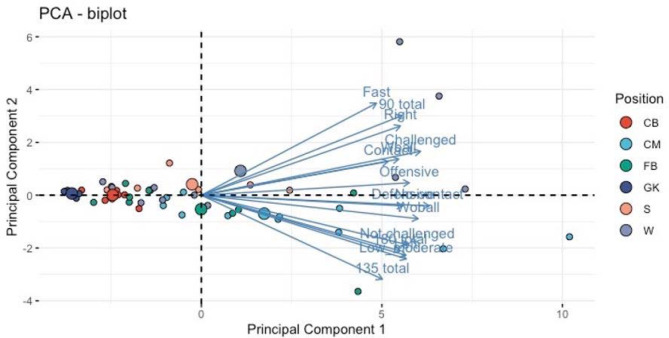
Biplot of PCs 1 and 2 with playing positions and contributions of different variables. Small dots = Relation of each individual player to the first two principal components. Large dots = Means of all the playing positions. Abbreviations: CB = centre back, CM = centre midfield, FB = full back, GK = goalkeeper, S = striker, W = winger, Wball = with a ball, Woball = without a ball

**Figure 3:**
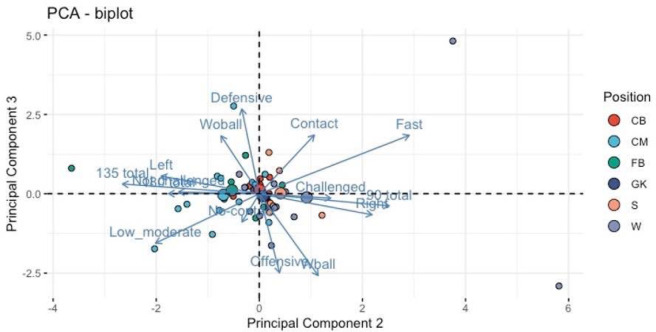
Biplot of PCs 2 and 3 with playing positions and contributions of different variables. Small dots = Relation of each individual player to the first two principal components. Large dots = Means of all the playing positions. Abbreviations: CB = centre back, CM = centre midfield, FB = full back, GK = goalkeeper, S = striker, W = winger

Biplots (Figures 2 and 3) show individual PC values for all players (small dots) and means per playing position (large dots). Contributions of the original variables are shown with blue arrows (a longer arrow indicating stronger contribution).

## Discussion

The aim of this study was to examine situational differences in 90° cuts, 135° cuts and 180° pivot turns in games with respect to different playing positions in youth soccer players during games. Previous studies have discovered differences between playing positions in physical demands and agility (Deprez et al., 2015; Granero-Gil et al., 2020). This is the first study to analyze the frequency of CODs commonly used in COD testing protocols in youth soccer games, including differences by player positions. Differences in physical loading between playing positions have been examined in previous studies (Barrett et al., 2018; Panduro et al., 2021) and the results of this study are similar taking into account lower demands for central defenders and the playing position’s specificity in physical demands in general (Bangsbo et al., 2006; Bradley et al., 2009, 2010). The first component, which explained most of the variation, was based on quantity of all initial variables. Centre midfield players performed more CODs during the game than others, regardless of the type of COD and the situation. Previous research has concluded that midfielders cover most distances during a game (O’Donoghue, 2002; Rienzi et al., 2000), perform less shuffling and are most involved in ball possession (Bloomfield et al., 2007), which would explain higher quantities of COD movements. The second principal component can be described as the attacking/goal-scoring opportunity-based component. It includes positive correlations with “fast”, “90° cuts” and to the “right”. Most of the players were right footed which could explain the dominance for cuts to the right within this component, since players would want to get their right leg open before shooting or crossing the ball in the attacking zone. The third component was related to reactive situations while defending, with positive correlations to “without a ball”, “fast”, “defensive” and “contact”. The second PC results are supported by previous research showing that strikers perform most high-intensity activity and are most often in contact situations (Bangsbo et al., 2006; Bloomfield et al., 2007; Di Salvo et al., 2007), while fullbacks and wingers are involved more often in high-speed situations than other positions (Bangsbo et al., 2006; Barrett et al., 2018).

Based on the means of PCA results, centre midfielders and wingers were involved in CODs more often than other positions, regardless of the situational pattern. Goalkeepers and centre backs, on the other hand, did not perform those types of CODs almost at all during games. Regarding more situational specific factors, centre midfielders and full backs performed more low and moderate speed CODs, 180° and 135° cuts, to the left and while not challenged by the opposition, whereas wingers and strikers completed more 90° cuts, to the right, at high speed being challenged by an opposing player. Winger and striker positions would therefore be involved more in attacking type COD situations than others. However, based on the analysis of the third component, defensive reactive situations were not based on playing positions *per se*, suggesting an influence of the opposition characteristics related to tactical formations and individual skills. This finding suggests that these situations should be practiced equally, regardless of the playing position. In general, our results indicate that practitioners could target testing, training, and physical preparation more effectively by considering playing position differences.

It should be noted that the count of CODs during games was highly related to the definition of a COD movement. Quantities of CODs in this study are in line with those found by Nedelec et al. (2014) and Baptista et al. (2018), but considerably lower than results from studies that included a broader definition of COD movements (Bloomfield et al., 2007; Granero-Gil et al., 2020; Morgan et al., 2022). Based on this fact, a more specific criteria-based definition of COD movements in soccer (and other evasion sports as well) is recommended, to increase the understanding of player loading and quality of COD analysis.

Differences with respect to specific playing positions have been studied earlier based on sprints and accelerations, and the results are in line with our findings on COD movements (Borghi et al., 2021). In both youth and adult soccer, wide midfield and wingback positions executed the highest number of high-speed accelerations and decelerations as well as sprints and high-speed running compared to other positions regardless of the playing formation. Centre midfielders performed more low speed accelerations and decelerations, but covered a greater distance compared to other positions, whereas centre defenders completed the least number of high and low speed movements (Barron et al., 2014; Oliva-Lozano et al., 2020). Patterns of activity vary during games due to playing positions: fullbacks and forwards cover greater full-speed running distances in U20 female high-level soccer than other positions (Ramos et al., 2017).

The results of our study can be valuable for planning specific training targeted at a particular playing position, agility testing and evaluation of readiness for return to play after injury. However, by means of video-analysis, it is difficult to determine demands related to velocity and deceleration of COD movements. This problem could be solved in future studies using inertial measurement units.

## Limitations

This study is not without limitations. Only one game per team was recorded and used for COD identification. There is an effect of the opponent on the team and individual player performance. In youth soccer, the opponent’s quality affects match performance – shots, sprints etc. (Varley et al., 2017). In this study, both female teams had “easier” games (4-0, 5-0 wins), whereas male teams played more challenging games (2-2 tie and 2-1 win), although all teams played against other tier 1 opponents in a league game. The field formation and specific tactics used by the coach can also have a great effect on player behaviour. Teams included in our study, being from the same club and having similar “game philosophy” and playing with a similar formation in all games, could bias our data and certainly affect generalizability. Playing time between the two halves was not equal between all the players and previous research has concluded that the number of CODs in youth soccer players might differ between the two halves (Morgan et al., 2022). In addition, players with lesser playing time might have played during a period in the game when the concentration of COD movements was lesser or higher. The sample size for PCA was not necessarily large enough (usually recommended to be minimum 100), but the amount of variables in relation to sample size was acceptable (Rojas-Valverde et al., 2020). However, as an analysis method, PCA tends to overfit the data especially with small sample sizes and thus, the generalizability of our results should be considered with caution (Osborne and Costello, 2019). Further studies with longer follow-up time, more games and addition of the opponent’s level and tactics used as variables, are recommended to increase understanding on this subject.

## Conclusions

Based on these results, particular playing positions affect the exposure to different types of COD movements during games in youth soccer, when looking at CODs performed in a similar way as they are commonly tested. The definition of COD in soccer is not very precise and ranges considerably based on the criteria used. Over 90° COD situations in youth soccer games are depended on the playing position and understanding these position specific demands can be helpful when testing youth players for performance or return to play readiness after injury. In addition, this information can assist in developing better testing protocols that meet gameplay demands of individual players. To increase the understanding and quality of future studies on COD movements, more comprehensive criteria, and a definition of change of direction movement is needed.
